# Influence of Harvest Time on the Chemical Profile of *Pereskia aculeate* Mill. Using Paper Spray Mass Spectrometry

**DOI:** 10.3390/molecules27134276

**Published:** 2022-07-02

**Authors:** Antonio Henrique de Souza, Henrique de Oliveira Prata Mendonça, Ana Cardoso Clemente Filha Ferreira de Paula, Rodinei Augusti, Camila Argenta Fante, Júlio Onésio Ferreira Melo, Lanamar de Almeida Carlos

**Affiliations:** 1 Agricultural Sciences Department, Federal University of São João del-Rei, Sete Lagoas 35701-970, MG, Brazil; antoniosouza@ufv.br (A.H.d.S.); hp.quimico@hotmail.com (H.d.O.P.M.); lanamar@ufsj.edu.br (L.d.A.C.); 2 Agricultural Sciences Department, Federal Institute of Educação, Ciência e Tecnologia de Minas Gerais, Bambui 38900-000, MG, Brazil; ana.paula@ifmg.edu.br; 3 Chemistry Department, Federal University of Minas Gerais, Belo Horizonte 31270-901, MG, Brazil; augusti.rodinei@gmail.com; 4 Food Department, Federal University of Minas Gerais, Belo Horizonte 31270-901, MG, Brazil; camilafante@ufmg.br

**Keywords:** phytochemicals, bioactive compounds, ora-pro-nobis, Barbados gooseberry, HPLC

## Abstract

This study evaluated the physicochemical characteristics and the production of bioactive compounds of *Pereskia aculeata* Mill. at different harvest times. Here, we performed a qualitative evaluation of the chemical profile by paper spray mass spectrometry (PSMS), the phenolic acid and flavonoid profile by high-performance liquid chromatography (HPLC), antioxidant activity, total carotenoids, total phenolic compounds, total flavonoids, total anthocyanins, color characteristics, total soluble solids (TSS), total solids (TS), pH, and total titratable acidity (TTA). The chemical profile was not affected, with the exception of 4,5-dimethyl-2,6-octadiene and azelaic acid, which was only identified in the leaves harvested during the winter. The content of four phenolic acids and three flavonoids were analyzed; out of these, no significant amounts of ellagic acid and quercetin were detected. There was no difference in production of bioactive compounds between seasons, reflecting the antioxidant activity, which also did not differ. Brightness, chroma, and leaf pH were the only physicochemical characteristics that did not vary between seasons.

## 1. Introduction

Ora-pro-nobis, otherwise known as the Barbados gooseberry (GBB) or *Pereskia aculeata* Mill. (Cactaceae), is a shrubby perennial plant, native to Tropical America and can reach a height of 10 m. It has long branches, prostrate, simple leaves with short petioles, and an elliptic, flat blade, fleshy texture, up to 12 cm long [1]. In some regions it is also known as leaf cactus, lemonvine, and locally in Brazil as ora-pro-nobis, azedinha, and lobrobó. It is favorable for cultivation due to being an easily propagated rustic plant [2]. *Pereskia aculeata* Mill. is used for various purposes, such as food, fodder, ornamental, and medicinal applications [3].

Bioactive compounds present in the GBB leaves (e.g., phenolics, carotenoids, vitamins A, C, and E), comprise the list of chemical constituents whose protective action in organisms is related to their antioxidant properties [4]. These phytochemical compounds are part of the secondary metabolism of plants, which can be affected by various factors, such as climate, photosynthetically active radiation (PAR), soil, and harvest time [5,6,7]. Some studies report the influence of harvest time on the production of bioactive compounds and the antioxidant activity of plants of commercial interest (e.g., eggplant, lettuce, tomato, and apricot) [8,9], however, for the species *Pereskia aculeata* Mill., further studies regarding this characteristic are needed.

Considering that bio-based components have attracted the attention of food industries, the GBB presents an interesting characteristic for the food industry. The mucilage present in its leaves and fruits has a high emulsifying potential, is rich in minerals, and contains all essential amino acids in its composition, which characterizes it as a nutritionally pleasing food ingredient [10]. Due to their anti-inflammatory and antinociceptive activities, GBB leaves may have an important role in the pharmaceutical industry. Recently, a phytotherapeutic cream was developed based on *P. aculeata* leaves and researchers tested its anti-inflammatory and anti-psoriatic potential, demonstrating the therapeutic relevance and marketing associated with the use of this species [11].

Collectively, these benefits justify the renewed interest for both the industrial purposes and consumption of this plant, however, it is important to provide information that contributes to its application, valuation, and rescue, in an attempt to fill knowledge gaps involving the evaluation and/or characterization of the chemical profile and content of bioactive compounds and antioxidant activity at different harvest times.

This study evaluated the influence of the harvest season (autumn and winter) on the chemical profile and the physical and chemical characteristics of the GBB leaves.

## 2. Results and Discussion

Here, it was reported for the first time how harvest time can influence the chemical profile, bioactive compound production and physicochemical characteristics in GBB leaves. To the best of our knowledge, many of the compounds shown in Table 1 have been detected for the first time in the leaves of this species.

A total of 56 substances of different chemical classes were detected in GBB leaves. The compounds 4,5-dimethyl-2,6-octadiene (*m*/*z* 138) and azelaic acid (*m*/*z* 187) were detected only in winter and were not detected in the leaves harvested in autumn. The stability of the chemical profile of GBB leaves when subjected to heat has already been demonstrated, as well as the detection of the presence of both compounds in fresh GBB leaves harvested in the same region, without mentioning the harvest season [16].

Caftaric acid is the main phenolic constituent of GBB leaf extract, accounting for more than 49% of its phenolic content, followed by the compounds quercetin-3-O-rutinoside (*m*/*z* 609) and isorhamnetin-O-pentoside-O-rutinoside (*m*/*z* 755), which account for 14.99 and 9.56% of the phenolic content of GBB leaves, respectively. Such compounds were found in both seasons the leaves were collected [1].

By employing high performance liquid chromatography (HPLC) analysis of the GGP leaves, it was possible to characterize the presence of phenolic acids, namely gallic acid, chlorogenic acid, caffeic acid, and ellagic acid. Based on the same principle, three flavonoids were quantified in the leaves, namely catechin, rutin, and quercetin, presented in Table 2.

Chlorogenic acid was the major phenolic compound detected in GBB leaves that was accounted in this study, in both the autumn (13.20 mg 100 g^−1^ dry sample) and winter (11.22 mg 100 g^−1^ dry sample) seasons.

Caffeic and chlorogenic acids, both found in significant concentrations in GBB leaves, inhibit the generation of reactive oxygen species, demonstrating the potential of GBB as an alternative source of phenolics, especially for poorer populations, since this plant, by being easy to grow, resistant to drought and frosts, adaptable to various types of soil, and being not demanding in terms of fertility, can be easily grown in several locations in the country [17].

Rutin was the majority flavonoid detected in GBB leaves in the autumn (9.27 mg 100 g^−1^ dry sample) and winter (12.22 mg 100 g^−1^ dry sample) seasons. This substance has therapeutic properties with antioxidant and anticarcinogenic potential, as well as having protective effects on the renal, cardiovascular, and hepatic systems [18].

Included in the PANC ground (*Plantas Alimentícias não convencionais*—Non-conventional food plants), GBB leaves are common in the regional cuisine of the state of Minas Gerais and has considerable nutritional value due to the high content of protein, iron, calcium, β-carotene, and the presence of ascorbic and folic acids, as well as other macro and micronutrients [16,19]. Caffeic and chlorogenic acids have cardioprotective and antioxidant properties [20]. Leaves of this species also contain phytochemicals, such as vitamin A (185.8 mg 100 g^−1^), vitamin C (1.4 mg 100 g^−1^), chlorogenic acid (up to 81.29 mg 100 g^−1^), p-coumaric acid (up to 5.39 mg 100 g^−1^), folic acid (19.3 mg 100 g^−1^), caffeic acid (6.41 mg 100 g^−1^), ferulic acid (2.15 mg 100 g^−1^), cis caftaric acid (9.5 ± 0.1 mg g^−1^), and quercetin (2.11 ± 0.02 mg g^−1^), that present antioxidant, antimicrobial, antinociceptive, and anti-inflammatory activities, helping in the prevention of several non-transmissible chronic diseases such as cancer and diabetes [1,4,13,21,22].

In addition, other classes of compounds detected in GBB leaves, show high medicinal potential, such as the substance procyanidin B1, which can suppress the replication of the virus that causes hepatitis C, besides being a potential candidate molecule against coronaviruses, including COVID-19 [23,24,25]. Furthermore, the phenolic constituents present in GBB leaves are possibly responsible for the indirect prebiotic action, highlighting their benefit in the consumption of this plant, which is often neglected [26].

The bioactive compound contents of GBB leaves collected in the autumn and winter are presented in Table 3.

There were no differences in the content of bioactive compounds in the leaves of GBB harvested in the autumn and winter (Table 3) that reflected in their antioxidant activity, which also showed no difference between one season and another. It was reported 2.66 g of GAE/kg of fresh matter in GBB leaves were harvested in the South region of Brazil during the winter, study, lower contents than those found in our work [22]. TPCs were high when compared to the content of these compounds quantified individually, possibly due to the fact that the estimate of total phenolic content includes all subclasses of compounds present in samples, encompassing both flavonoids and non-flavonoids, such as tannins, for example [27].

It was reported that TF contents near to 680 mg 100 g^−1^ sample on dry base and TA contents near to 56 mg cyanidin-3-glucoside 100 g^−1^ sample on dry base for the leaves of *Stachys byzantina* were observed, which is also a leafy PANC collected at the same site [28]. The TF contents of 1215.90 ± 201.35 mg 100 g^−1^ sample on dry base and a TA of 117.23 ± 19.32 mg cyanidin-3-glycoside 100 g^−1^ sample on dry base were previously presented for *in natura* GBB leaves collected at the same site [16].

The factors such as location, cultivation system, plant age at harvest, as well as the variability that may occur within the species itself, can influence the centesimal composition of leafy PANC [29]. Thus, it was assessed that the bioactive composition of GBB may also be affected by such factors, since there are differences between the contents described here and what has been reported in other cited works.

### Physical–Chemical Characteristics

GBB leaves harvested in the fall showed significant differences (*p* < 0.05) for the parameters °Hue, TSS, TS, and TTA when compared to the material collected in the winter. The parameters brightness, chroma, and pH showed no significant difference.

In three-dimensional color space, L* indicates the shade of the leaves and varies from 0 (black) to 100 (white), and the closer to 100, the lighter the leaf, that is, the greater the light reflectance capacity of the leaf. As shown in Table 4, the values of L* did not vary between one season and another.

In previous studies, luminosity values close to 47 for GBB leaves were found, values that are close to what was reported here, corroborating what was shown in our work [30].

Evaluating the stability of bioactive compounds during the storage of goldfish leaves, which are also considered an PANC, reported L* values near 56 for leaves collected in the same region. Such values are higher than the values we found for GBB, which is explained by the characteristic, white-colored hairiness in the leaves of this vegetable, which promote its higher L* [31].

C* indicates the color saturation, where close to zero the colors are neutral, and close to 60 the colors are vivid, emphasizing the greater intensity in the color of the vegetable leaves the closer these values are to 60 [32]. In both harvest seasons, the leaves of GBB showed close saturation values (11.59 ± 1.72 in autumn and 10.79 ± 3.26 in winter).

°Hue values indicate the real color of the vegetable leaf and in both seasons, they were between 90 and 180°, corresponding to the second color quadrant that goes from yellow to green. GBB leaves harvested in autumn showed °Hue of 115.48 ± 2.05, which was higher (*p* < 0.05) than leaves collected in winter (111.70 ± 3.52). A value close to 122.5° was reported for GBB leaves [30].

The TSS contents of 7.37 ± 0.30 °Brix for *in natura* GBB leaves collected in the same location have already been found [16]. It was reported that 12.46 ± 0.47% ST in *Pereskia aculeata* leaves were collected during the spring season in the city of São Gonçalo do Abaeté (MG) [33], values close to what was reported in this work, as shown in Table 5.

Biomass provides the most direct assessment of plant performance, in terms of growth. Therefore, changes in biomass can be translated into the most comprehensive indicator of a plant’s ability to respond to and benefit from various resource supplies, such as water, light, CO_2_, and nutrients [34]. Leaves of GBB harvested in winter showed a higher dry mass when compared to the leaves harvested in the fall season, showing a higher carbon accumulation performance of this vegetable in this season.

As for pH values, GBB leaves showed a low acidity, as to be expected for most leafy vegetables. Souza et al. [16] reported equal pH values in GBB leaves harvested in the same location during (5.07 ± 0.28). As for TTA, the same study reported values of 0.11 ± 0.0066 citric acid 100 g^−1^ of fresh sample.

## 3. Materials and Methods

### 3.1. Samples

GBB leaves (*Pereskia aculeata* Mill.), were collected from the HNC Bank of the Empresa de Pesquisa Agropecuária de Minas Gerais (EPAMIG), Prudente de Morais-MG (19°27′13.9″ S 44°09′25.7″ O), Brazil, in two distinct seasons, on 25 April 2018 (autumn) and on 6 August 2018 (winter) in the morning period. The exsiccate of said species is deposited in the herbarium of EPAMIG with registration PAMG 57026. Cultivation was performed without the use of pesticides and the cultural treatments were carried out according to the development of the plants.

### 3.2. Paper Spray Mass Spectrometry Chemical Profile

Chemical profile analysis of the samples was performed using a LCQ Fleet mass spectrometer (Thermo Scientific, San Jose, CA, USA) equipped with a paper spray ionization source type. It was performed in triplicate in positive and negative ionization modes [35]. In this analysis, the chromatographic paper was cut into an equilateral triangle shape (1.5 cm) and positioned at the entrance of the mass spectrometer. A metal connector was used to support the equilateral chromatographic paper and positioned 0.5 cm apart with the aid of a mobile platform (XYZ).

A high voltage source was provided by the mass spectrometer through a copper wire attached to the contraption. Finally, 2.0 µL of the sample was applied to the edge of the equilateral chromatographic paper, 40.0 µL of methanol was applied to the paper, and the voltage source was connected for data acquisition. For the analyses, the equipment was operated at a voltage of +4.0 kV for the positive ionization mode and −3.0 kV for the negative ionization mode; a capillary voltage of 40 V; transfer tube temperature was maintained at 275 °C; tube lens voltage of 120 V; and a mass range of 100 to 1000 *m*/*z* for both positive and negative ionization mode. Ions and their acquired fragments were identified according to data described in the literature. A range of collision energies from 15 to 45 eV was used to fragment the compounds [35].

Analysis of each sample was performed in triplicate and the presence of the compound was taken into consideration when it appeared at least twice, it was absent when it did not appear, and/or when it appeared only once in the triplicate.

### 3.3. Phenolic Acid and Flavonoid Profile

Separation and quantification of phenolic acids and flavonoids were performed by high-performance liquid chromatography (HPLC) in an ultra-high performance liquid chromatograph (Waters, Acquity UPLC^®^ Class, Milford, MA, USA) equipped with a diode array UV detector, quaternary pump, online degasser, autosampler, and an Acquity UPLC^®^ BEH C18 (2.1 × 100 mm; 1.7 µm, Waters, Milford, MA, USA). The run was isocratic, with the mobile phase consisting of acetonitrile (A) and water with formic acid 0.25%, (ratio 5:95) under a constant flow of 0.3 mL min^−1^ with a sample injection volume of 1 µL [36]. Concentrations were calculated by their comparison with the curves of the external standards in the range 12.5 to 260 µg mL^−1^. Calibration curves were established and based on the linear correlation between the concentration of the standards and the area of the peaks, corresponding to the individual acids and flavonoids. Results are expressed as mg per 100 g of sample on a dry base.

### 3.4. Total Carotenoids (TC)

TC content was determined by spectrophotometry at a 450 nm wavelength. Carotenoids were extracted by partitioning, with an application of petroleum ether and acetone [37]. Results are expressed as milligrams of carotenoids per 100 g of sample on a dry base.

### 3.5. Total Phenolic Compounds (TPC)

TPC content was determined by the Folin–Ciocalteau method with the comparison of a calibration curve constructed with gallic acid [38]. Absorbance was read in a spectrophotometer (FEMTO) at 740 nm. The results are expressed as grams of gallic acid equivalents (GAE) per 100 g of sample on a dry base.

### 3.6. Total Flavonoids (TF)

Analysis of total flavonoid and content was performed following a method already described [39]. Absorbance was read in a spectrophotometer (FEMTO, 700S) at 374 nm. Results are expressed as milligrams of flavonoids per 100 g of sample on a dry basis.

### 3.7. Total Anthocyanins (TA)

Analysis of total anthocyanin content was performed following a method already described [38]. Absorbance was read in a spectrophotometer (FEMTO, 700S) at 535 nm. Results are expressed as mg cyanidin-3-glycosidium (CG) per 100 g of sample on a dry base.

### 3.8. Antioxidant Activity (TAA)

The evaluation of antioxidant activity was performed by the photocolorimetric method of the stable free radical DPPH (1,1-diphenyl-2-picrylhydrazyl) [40], which is based on the sequestration of the 1,1-diphenyl-2-picrylhydrazyl radical (DPPH) by antioxidants, which produces a decrease in absorbance at 517 nm. Results are expressed as µmol of trolox equivalent per gram of sample on a dry base.

### 3.9. Physical–Chemical Characteristics

The instrumental color analysis was performed with a colorimeter (Konica Minolta, CR-410, Tokyo, Japan) in a three-dimensional color space L*, C*, h where: L* = Luminosity, C* = Chroma, and h = Hue angle (°Hue). After calibration of the equipment using the white plate CRA43, readings were made in three distinct points on the surface of each leaf, in order to obtain an average value. Total soluble solids (TSS) levels were determined according to the methodology already described [41]. Samples were crushed, filtered, and placed on a prism of a digital refractometer (Reichert, R2 Mini Digital Pocket Refractometer, Depew, NY, USA) with internal temperature compensation. Results were expressed in °Brix.

For the quantifying of total solids (TS), about 2 g of the homogenized samples were subjected to a temperature of 105 °C in a muffle (FANEM, 515) to sterilization and drying oven for 48 h. The percentage of ST was obtained by the difference between the initial and final masses (after the muffle) of the samples [41]. The value of pH was determined by potentiometry with the aid of a pH meter (Tekna digital, T-1000), through direct immersion of the electrode in the homogenized sample and with the addition of 50 mL distilled water [15]. For total titratable acidity (TTA) determination, a 0.01 N NaOH solution was used as a standard and phenolphthalein as an indicator, with measurement assistance using a pH meter. Results were expressed as the percentage of citric acid [41].

### 3.10. Statistical Analysis

An entirely randomized design was used, with 5 repetitions. Evaluations were performed in triplicate. Assumptions of normality and homogeneity of variances were verified by the Shapiro–Wilk and Levene tests. Data from all analyses were submitted to an analysis of variance (ANOVA) and means were compared using the Tukey’s test with 5% probability (*p* < 0.05), using the software R [42].

## 4. Conclusions

No differences were detected in the chemical profile of the GBB leaves collected in the autumn and winter seasons, except for the production of the compounds 4,5-dimethyl-2,6-octadiene (*m*/*z* 138) and azelaic acid (*m*/*z* 187) in winter, but not in autumn.

The presence of ellagic acid and quercetin flavonoid was not detected in GBB leaves, regardless of the harvest season. In both seasons, chlorogenic acid and the flavonoid rutin were the compounds found in the highest amounts in GBB leaves.

Carotenoid content, total phenolics, flavonoids, and anthocyanins were not influenced by the harvest time, which reflected in the antioxidant activity, which also showed no significant difference.

The characteristics °Hue and TTA presented higher values in the autumn, while the TSS and TS were higher during the winter. These variations may be related to edaphoclimatic factors involving the amount of rainfall, temperature, photosynthetically active radiation, and humidity, factors that can favor or disadvantage a vegetable in certain environmental conditions.

GBB appears to be a potential source of bioactive compounds, with antioxidant properties, and is present in the classes of carotenoids (TC) and phenolic compounds (TPC, TF and TA). This is unprecedented information on the chemical profile of Ora-pro-nobis at different harvest times, provided by paper spray.

Information on the content of these phytochemicals in the composition of PANC can contribute in a relevant way to the rescue of PANC, which are part of the culture of some traditional populations, besides pointing out new options for the food and pharmaceutical industries. In addition, the wide variety of vegetables in which these compounds can be found, generates a gradient of options that can be used as sources of nutrients without always sticking to the same conventional vegetables, also being of extreme importance for the pharmaceutical industry, which uses these compounds for the production of medicines.

## Figures and Tables

**Table 1:** Qualification of the chemical profile of GBB in the autumn and winter season by PSMS.

No.	Tentative Identification	Formula	*m*/*z*	MS/MS	Reference	Season	
						Autumn	Winter
1	Malic acid	C_4_H_6_O_5_	133	115	Cabañas-García et al. [12]	X	X
2	Protocatechuic aldehyde	C_7_H_6_O_3_	137	121, 109	Cabañas-García et al. [12]	X	X
3	4,5-dimethyl-2,6-octadiene	C_10_H_18_	138	123, 95, 69, 41	Pinto et al. [13]	-	X
4	Dihydroxy methoxy butanoic acid	-	149	135, 131, 119, 103	Cabañas-García et al. [12]	X	X
5	Caffeic acid	C_9_H_8_O_4_	179	163, 135, 109	Cabañas-García et al. [12]	X	X
6	1-Methoxy-*p*-toluyl-propan-2-ol	-	181	144, 116, 103, 87, 73	Pinto et al. [13]	X	X
7	Azelaic acid	C_9_H_16_O_4_	187	169, 125	Cabañas-García et al. [12]	-	X
8	Quinic acid	C_7_H_12_O_6_	191	173, 111, 93, 85	Abdul Rahman et al. [14]	X	X
9	Ferulic acid	C_10_H_10_O_4_	193	149, 134	Jiménez-Aspee et al. [15]	X	X
10	Sebacic acid	C_10_H_18_O_4_	201	185, 157	Cabañas-García et al. [12]	X	X
11	Nerolidol	C_15_H_26_O	222	204, 161, 93, 69, 41	Pinto et al. [13]	X	X
12	Synapic acid	C_11_H_12_O_5_	223	208, 179, 164	Cabañas-García et al. [12]	X	X
13	*p*-Hydroxynonanophenone	-	233	219, 167, 135, 121	Cabañas-García et al. [12]	X	X
14	Syringinic Acid Acetate	-	239	197, 195, 179, 149, 135, 107	Cabañas-García et al. [12]	X	X
15	Isomer of piscidic acid	-	255	193, 165, 135, 119, 107	Cabañas-García et al. [12]	X	X
16	Palmitic acid	C_16_H_32_O_2_	256	213, 129, 73, 60, 43	Pinto et al. [13]	X	X
17	Methyl Palmitate	C_17_H_34_O_2_	270	227, 143, 87, 74, 55, 43	Pinto et al. [13]	X	X
18	Butein	-	271	163, 137, 135, 121, 108	Cabañas-García et al. [12]	X	X
19	Neophytadiene	C_20_H_38_	278	123, 95, 68, 43	Pinto et al. [13]	X	X
20	Kaempferol	C_15_H_10_O_6_	285	255, 229, 151	Abdul Rahman et al. [14]	X	X
21	Dalbergioidin	C_15_H_12_O_6_	287	217, 179, 165, 163, 147, 125, 109	Cabañas-García et al. [12]	X	X
22	Catechin	C_15_H_14_O_6_	289	271, 245, 151, 137	Abdul Rahman et al. [14]	X	X
23	Linolenic acid methanoate	C_19_H_32_O_2_	292	108, 95, 79, 67, 55, 41	Pinto et al. [13]	X	X
24	Nordihydrocapsiate	C_17_H_26_O_4_	293	277, 263, 247, 157, 153, 141	Cabañas-García et al. [12]	X	X
25	Caffeic acid derivative	-	295	179, 163, 133	Garcia et al. [1]	X	X
26	Phytol	C_20_H_40_O	297	123, 95, 81, 71, 57, 43	Pinto et al. [13]	X	X
27	Methyl Octadecanoate	C_19_H_38_O_2_	298	143, 87, 74, 55, 43	Pinto et al. [13]	X	X
28	Quercetin	C_15_H_10_O_7_	301	273, 179, 151	Abdul Rahman et al. [14]	X	X
29	3,5-Dihydroxy-4-methyloxolan-2-yl methoxy-6-hydroxymethyl oxane-3,4,5-triol	-	309	293, 279, 147, 131	Cabañas-García et al. [12]	X	X
30	Cis-Caphthalic Acid	C_13_H_12_O_9_	311	179, 149	Garcia et al. [1]	X	X
31	Hexoside of protocatechuic acid	-	315	255, 211, 153, 137, 121, 109	Cabañas-García et al. [12]	X	X
32	Tricosan	C_23_H_48_	324	113, 99, 85, 71, 57, 43	Pinto et al. [13]	X	X
33	Fertric Acid	C_14_H_14_O_9_	325	193, 179, 163	Cabañas-García et al. [12]	X	X
34	Thianshic acid	C_18_H_34_O_5_	329	165, 127	Cabañas-García et al. [12]	X	X
35	Monogaloyl glucose	C_13_H_16_O_10_	331	271, 211	Abdul Rahman et al. [14]	X	X
36	Plastoquinone 3	C_23_H_32_O_2_	339	203, 163, 149, 135	Cabañas-García et al. [12]	X	X
37	1-Caffeoylquinic Acid	-	353	191, 179, 135, 93	Abdul Rahman et al. [14]	X	X
38	Vaccihein A	C_18_H_18_O_9_	377	361, 347, 319, 289, 125	Cabañas-García et al. [12]	X	X
39	9,10-dihydroxy-4,7-megastigmadien-3-one hexoside	-	385	223, 205	Jiménez-Aspee et al. [15]	X	X
40	Campesterol	C_28_H_48_O	400	382, 367, 315, 289, 213, 145, 43	Pinto et al. [13]	X	X
41	Stigmasterol	C_29_H_48_O	412	351, 271, 255, 159, 133, 83, 69, 55	Pinto et al. [13]	X	X
42	Sitosterol	-	414	396, 329, 303, 213, 145, 107, 81, 57, 43	Pinto et al. [13]	X	X
43	Taraxerol	C_30_H_50_O	426	302, 287, 204, 135, 95, 69	Pinto et al. [13]	X	X
44	Quercetin-*O*-pentoside	-	433	300, 255, 151	Abdul Rahman et al. [14]	X	X
45	Lucuminic Acid	C_19_H_26_O_12_	445	163, 119, 107	Cabañas-García et al. [12]	X	X
46	Kaempferol glycoside	-	447	284, 256, 151	Abdul Rahman et al. [14]	X	X
47	Quercetin-3-glucoside	C_21_H_20_O_12_	463	300, 179, 151	Abdul Rahman et al. [14]	X	X
48	Isorhamnetin 3-*O*-β-glucoside	-	477	315	Jiménez-Aspee et al. [15]	X	X
49	Procyanidin B1	C_30_H_26_O_12_	577	451, 425, 407, 289, 161, 137, 125	Abdul Rahman et al. [14]	X	X
50	Kaempferol-3-*O*-rutinoside	C_27_H_30_O_15_	593	285	Garcia et al. [1]	X	X
51	Quercetin-*O*-pentosid-O-hexoside	-	595	463, 301	Garcia et al. [1]	X	X
52	Quercetin-3-*O*-rutinoside	C_27_H_30_O_16_	609	301	Garcia et al. [1]	X	X
53	Isorhamnetin-3-*O*-rutinoside	-	623	315	Garcia et al. [1]	X	X
54	Quercetin-*O*-pentosid-*O*-rutinoside	-	741	609, 301	Garcia et al. [1]	X	X
55	Isorhamnetin-*O*-pentosid-*O*-rutinoside	-	755	623, 315	Garcia et al. [1]	X	X
56	Isorhamnetin dirhamnoside hexoside	-	769	315	Jiménez-Aspee et al. [15]	X	X

X indicates the presence of the compound in the sample.

**Table 2:** Mean values (expressed in mg 100 g^−1^ of sample, on dry base) of gallic acid, chlorogenic acid, caffeic acid, ellagic acid, catechin, rutin, and quercetin contents of *Pereskia aculeata* Mill. leaves in the autumn and winter seasons.

Phenolic Acids and Flavonoids	Season	
	Autumn	Winter
Gallic acid	0.62	0.47
Chlorogenic acid	13.20	11.22
Caffeic acid	10.18	5.72
Ellagic acid	^nd^	^nd^
Catechin	5.47	2.39
Rutin	9.27	12.22
Quercetin	^nd^	^nd^

^nd^ = not detected.

**Table 3:** Mean values ± standard deviation of contents of TC (mg 100 g^−1^ dry sample), TPC (in g GAE 100 g^−1^ dry sample), TF (in mg 100 g^−1^ dry sample), TA (in mg cyanidin-3-glucoside 100 g^−1^ dry sample), and TAA (in µmol ET/g dry sample) of *Pereskia aculeata* Mill. leaves in the autumn and winter seasons.

Season	Bioactive Compounds and Antioxidant Activity				
	TC	TPC	TF	TA	TAA
Autumn	201.05 ± 29.09 ^a^	10.00 ± 1.24 ^a^	804.00 ± 78.01 ^a^	79.21 ± 5.27 ^a^	1345.22 ± 46.24 ^a^
Winter	207.83 ± 10.56 ^a^	10.50 ± 1.43 ^a^	818.35 ± 84.00 ^a^	72.36 ± 4.79 ^a^	996.31 ± 30.00 ^a^
CV (%)	19.6	8.38	4.77	21.28	8.67

CV = coefficient of variation. Means followed by the same letter in the columns do not differ significantly from each other at the 5% significance level by the Tukey’s test.

**Table 4:** Mean values ± standard deviation of instrumental color parameters L*, C*, and °Hue of *Pereskia aculeata* Mill. leaves in the autumn and winter seasons.

Season	Parameter		
	L*	C*	°Hue
Autumn	48.32 ± 1.54 ^a^	11.59 ± 1.72 ^a^	115.48 ± 2.05 ^a^
Winter	48.28 ± 2.31 ^a^	10.79 ± 3.26 ^a^	111.70 ± 3.52 ^b^
CV (%)	3.63	2.07	1.67

CV = coefficient of variation. Means followed by the same letter in the columns do not differ significantly from each other at the 5% significance level by the Tukey’s test.

**Table 5:** Mean values ± standard deviation of the TSS (°Brix), TS (%), pH, and TTA (g citric acid 100 g^−1^ of fresh sample) parameters of *Pereskia aculeata* Mill. leaves in the autumn and winter seasons.

Season	Parameter			
	TSS	TS	pH	TTA
Autumn	4.74 ± 1.54 ^b^	12.41 ± 1.29 ^b^	5.18 ± 0.26 ^a^	0.30 ± 0.049 ^a^
Winter	7.79 ± 0.34 ^a^	16.76 ± 1.29 ^a^	5.12 ± 0.27 ^a^	0.12 ± 0.023 ^b^
CV (%)	19.6	8.38	4.77	21.28

CV = coefficient of variation. Means followed by the same letter in the columns do not differ significantly from each other at the 5% significance level by the Tukey’s test.

## Data Availability

Not applicable.
